# Community acceptability of use of rapid diagnostic tests for malaria by community health workers in Uganda

**DOI:** 10.1186/1475-2875-9-203

**Published:** 2010-07-13

**Authors:** David Mukanga, James K Tibenderana, Juliet Kiguli, George W Pariyo, Peter Waiswa, Francis Bajunirwe, Brian Mutamba, Helen Counihan, Godfrey Ojiambo, Karin Kallander

**Affiliations:** 1 Department of Epidemiology and Biostatistics, Makerere University School of Public Health, P.O. Box 7072, Kampala, Uganda; 2 Division of Global Health, IHCAR, Department of Public Health Sciences, Karolinska Institutet, SE 17177 Stockholm, Sweden; 3 The African Field Epidemiology Network, plot 23, Mackenzie Vale, Kololo, P.O. Box 12874, Kampala, Uganda; 4 Malaria Consortium Africa, Plot 2, Sturrock Road, P.O. Box 8045, Kampala, Uganda; 5 Department of Community Health and Behavioral Sciences, Makerere University School of Public Health, P.O. Box 7072, Kampala, Uganda; 6 Department of Health Policy, Planning and Management, Makerere University School of Public Health, P.O. Box 7072, Kampala, Uganda; 7 Mbarara University of Science and Technology, P.O. Box 1410, Mbarara, Uganda

## Abstract

**Background:**

Many malarious countries plan to introduce artemisinin combination therapy (ACT) at community level using community health workers (CHWs) for treatment of uncomplicated malaria. Use of ACT with reliance on presumptive diagnosis may lead to excessive use, increased costs and rise of drug resistance. Use of rapid diagnostic tests (RDTs) could address these challenges but only if the communities will accept their use by CHWs. This study assessed community acceptability of the use of RDTs by Ugandan CHWs, locally referred to as community medicine distributors (CMDs).

**Methods:**

The study was conducted in Iganga district using 10 focus group discussions (FGDs) with CMDs and caregivers of children under five years, and 10 key informant interviews (KIIs) with health workers and community leaders. Pre-designed FGD and KII guides were used to collect data. Manifest content analysis was used to explore issues of trust and confidence in CMDs, stigma associated with drawing blood from children, community willingness for CMDs to use RDTs, and challenges anticipated to be faced by the CMDs.

**Results:**

CMDs are trusted by their communities because of their commitment to voluntary service, access, and the perceived effectiveness of anti-malarial drugs they provide. Some community members expressed fear that the blood collected could be used for HIV testing, the procedure could infect children with HIV, and the blood samples could be used for witchcraft. Education level of CMDs is important in their acceptability by the community, who welcome the use of RDTs given that the CMDs are trained and supported. Anticipated challenges for CMDs included transport for patient follow-up and picking supplies, adults demanding to be tested, and caregivers insisting their children be treated instead of being referred.

**Conclusion:**

Use of RDTs by CMDs is likely to be acceptable by community members given that CMDs are properly trained, and receive regular technical supervision and logistical support. A well-designed behaviour change communication strategy is needed to address the anticipated programmatic challenges as well as community fears and stigma about drawing blood. Level of formal education may have to be a criterion for CMD selection into programmes deploying RDTs.

## Background

Globally 3.2 billion people remain at risk of malaria and nearly one million malaria deaths occur each year, mostly in children under five years of age in sub-Saharan Africa [1]. Besides neonatal-related causes, malaria is the second leading cause of morbidity and mortality in Africa, and accounts for 21-26% of all under-five mortality in Uganda [2,3]. Many of these deaths occur at home due to poor access to health care, inappropriate or delayed care seeking and inadequate quality of health services [2,4]. Based on the current body of evidence, community health workers (CHWs) can play an important role in increasing coverage of essential interventions for child survival [5]. One such strategy recommended by WHO and UNICEF is Home Management of Malaria (HMM) which has been shown to be effective in reducing malaria mortality and morbidity in under-five children in a number of malaria endemic countries [6-8].

Since 2002, Uganda has adopted and implemented HMM at scale, locally referred to as Home Based Management of Fever (HBMF) strategy [9-11]. Under HBMF, community medicine distributors (CMDs) provided unit-dosed prepackaged Homapak™ (chloroquine and sulphadoxine/pyrimethamine - CQ + SP) presumptively to children that presented with, or had a history of fever [9]. More than 45% of first care contact for under-five children with fever was obtained through HBMF in program areas [12], and Kilian *et al *[13] found that caregivers of children had high acceptability towards Homapak™.

Due to increase in resistance to CQ and SP, in 2006 the National Malaria Control Programme (NMCP) made a policy change to cost-free artemisinin-based combination therapy (ACT) at all levels of the health system - including community level. Coartem^®^, the ACT that is in use Uganda is composed of a fixed combination of 20 mg of artemether and 120 mg of lumefantrine and is supplied in prepacked weight- and age-specific forms. There are concerns that the introduction of such a highly efficacious but expensive new treatment at community level, with reliance on presumptive diagnosis alone, may lead to excessive use, increased costs and risk of development of resistance [14,15].

The encouraging decline in malaria prevalence that has been observed in several African countries where integrated malaria control strategies have been implemented on a large scale [16-20] may also amplify the risk of misdiagnosis of fever episodes in absence of malaria diagnostic tools [21]. With policies that recommend presumptive treatment of fever, health workers and caretakers are less likely to look for other causes of fever, leading to delay in appropriate treatment and higher case fatality rates among non-malaria fevers than in malaria fevers [22]. There is limited evidence that shows change in malaria prevalence in the recent past in Uganda [23].

Given that rapid diagnostic tests (RDTs) are now available with sensitivities comparable to routine microscopy in detecting malaria [24-27] these tests are now increasingly being seen as an alternative to improve diagnosis and quality of care of febrile children in malarious areas [10,28]. RDTs are now being rolled out in many countries to all tiers of the formal health care system to guide malaria treatment decisions in routine care [29]. The potential role of RDTs in HMM is still highly debated; a facility based study in Tanzania found some caregivers with reservations about RDTs for malaria, thinking they were HIV test kits [30]. Studies from sub-Saharan Africa [31-33] document community stigma about drawing blood that may impede acceptability of RDTs. In addition, English *et al *[34] argue that there is inadequate evidence to support abandoning presumptive treatment and that African health systems have yet to demonstrate the capacity to support a shift toward laboratory-confirmed diagnosis rather than presumptive treatment of malaria in children under five. Some of these concerns include the implications of not providing treatment to childern with false-negative test results. Finally, diffusion of innovations in the health system depend on individuals' perceptions of the innovation, characteristics of the individual who may adopt the change, and contextual factors within the community [35]. It is still unclear what role RDTs will play in a situation where HBMF recommendations are blended with local and biomedical knowledge, and little is known about whether the community will accept the use of RDTs in the hands of CMDs with low education level and no formal health care background. The aim of this study was, therefore, to explore community acceptability of the use of RDTs for malaria diagnosis by CMDs within the context of community case management of fever in children under five in Uganda.

## Methods

### Study area

The study was conducted in the rural Ugandan district of Iganga as part of a larger study on the feasibility of deploying RDTs at community level in Uganda (Clinical Trials.gov Identifier NCT00720811). Uganda has an estimated population of 34 million inhabitants of whom about 80% live in rural areas. The economy is predominantly agricultural with the majority of the population dependent on subsistence farming.

Iganga District is located in South Eastern Uganda, approximately 112 km from Kampala, the capital city of Uganda. Iganga district is served by a 200-bed capacity hospital, and 81 health centres at county, sub-county and parish level. The main local language spoken in the district is Lusoga. Iganga has high transmission rates (holoendemic) for malaria [36]. HBMF was started in Iganga district in 2003 with district-wide 3-day training of CMDs in use of Homapak^®^. All the CMDs were retrained over 3 days in using Coartem^® ^in 2006, but due to inadequate stocks of ACT at national level the medicines were limited to facility-based deployment and were not deployed through CMDs during this study period. The study was conducted in August 2008 in the sub-county of Namungalwe, which has a total population of 32,911 in seven parishes and 19 villages. There are a total of 48 CMDs in the sub-county, majority of who are female and have attained some secondary school education. The sub-county of Namungalwe was selected because of a planned intervention study that will introduce the use of RDTs by CMDs.

### Data collection

A qualitative research approach using Focus Group Discussions (FGDs) and Key Informant (KI) interviews was deemed appropriate for the study aim. Ten FGDs with 6-10 participants in each group were conducted with caregivers of under-five children and CMDs (Table 1). Whereas most fevers are managed by mothers, FGDs were also conducted separately with fathers, as they play a significant role in the health care seeking of sick children, especially when costs are incurred [37].

**Table 1:** Number and mix of key informants and focus groups conducted

Key Informant Interviews (KIIs)	Number	Focus Group Discussions (FGDs)	Number
Health workers at local Health centre III	2	Female caregivers/mothers of children under five years	4

Civil society leaders/teachers	2	Male caregivers/fathers of children under five years	4

Local political leaders	2	Community medicine distributors (CMDs)	2

Health unit management committee members	2		

Religious leaders	2		

FGD participants were mobilized by the community leaders and purposively selected if they had ever cared for a child with fever. FGDs were conducted in different parishes, and took place in one of the homes of the participants to provide for privacy. Pre-designed interview guides were piloted and used to collect information on trust and confidence in CMDs, community willingness and acceptability of CMDs to use RDTs, anticipated challenges that CMDs could be faced with using RDTs, and stigma associated with taking blood from children in the community. Both the interviewer and the note taker were social scientists, spoke both English and the vernacular fluently and were experienced in conducting FGDs. The first FGDs were attended by two of the research team members who reviewed the notes afterwards. They also discussed with the interviewer and the note taker their experiences of the emerging issues and whether questions were being understood.

Key informant interviews were conducted with health workers and different community leaders (Table 1) to capture their experiences of CMD management of sick children, their perceptions of CMDs' abilities in using RDTs as part of the treatment of children with fever, anticipated challenges that CMDs could face with using RDTs and stigma associated with taking blood from children in the community. The research assistants were trained by the investigators on how to use the KI interview guide.

All the FGDs and KI interviews were conducted in the local language, transcribed and later translated into English by the interviewers. The participants had never seen nor had an RDT used on them, and during interviews the RDT test was described to the interviewees.

### Data analysis

Manifest content analysis [38] was used to categorize key issues out of the data. The unit of analysis was the transcripts from FGDs and KIIs. The authors (DM, JT, JK, BM, GO and KK) read through the data, identified different issues and debated them, and eventually developed codes. A second review of the material was done that generated more codes, which were discussed and agreed upon. These codes were merged into categories and then into themes reflecting the study objectives and other emerging issues.

### Ethical clearance

Ethical approval for the study was obtained from the WHO Ethical Review Committee, the Makerere University School of Public Health Institutional Review Board, and the Uganda National Council for Science and Technology. Presentations to the district health team for Iganga district, as well as to the local area authorities at the sub-county were made and permission obtained from them to conduct the study. Individual written (signed or thumb print) informed consent was obtained from each of the study participants for both key informant interviews and focus group discussions.

## Results

There were four key findings of the study: (1) CMDs are trusted by their communities because of their commitment to voluntarism, accessibility, and the perceived effectiveness of the anti-malarial drugs used, and their activities have led to a perception of reduced workload among health workers in health facilities; (2) community members, health workers and CMDs welcomed the use of RDTs by CMDs provided they have sufficient education level, are trained in their use, and supported to follow up children; (3) fears were expressed that CMDs who collect blood using RDTs could expose the children to HIV, that the tests could be used to test children for HIV, or that the blood could be used for witchcraft; (4) CMDs were anticipated to face challenges with transport for follow up of patients and re-stocking of supplies, adults demanding to be tested, and caregivers insisting their children be treated instead of being referred.

### Trust in and acceptability of CMDs managing children with fever

The role of CMDs in malaria management was well appreciated by the community since the CMDs were perceived to be accessible both physically and socially. Many community members reported trust and confidence in CMDs ability to handle sick children given their past experiences of helpful available CMDs.

*"She [the CMD] even asks us to take our children when they are sick to give us drugs. She has been very good because even if I go to her place at night she opens and gives me drugs. Even if I find her in the garden digging she comes back home to give me medicine. I trust her." *(FGD, female caregivers)

The spirit of volunteerism as demonstrated by CMDs not demanding to be paid for their services was greatly appreciated by community members, and their trust and confidence in the CMD services was often the main motivating factor for the CMDs to do voluntary work. The community members who had used the CMDs perceived the medicines provided to them as effective in treating children with malaria, and the recovery of sick children after treatment by CMDs was said to enhance the reputation of the CMDs amongst their neighbours and community.

*"According to me, they [CMDs] have done commendable work. They have a good relationship with the people and they have helped them a lot. They have been effective in distributing Homapak*^® ^*and Coartem*^®^*. I heard that the tablets are to be distributed freely and they are doing it - they fulfilled the intention. My twins are making 3 years and I've never taken them to hospital. They are treated at home and given Coartem*^®^*. They [CMDs] have never asked for money for treatment." *(KI, community leader)

Health workers reported to have observed a reduced workload at the health facilities as a result of CMD management of children with fever in the communities.

*"We have trained CMDs in many workshops. We taught them about the danger signs, so in case of such they refer them to the health centre immediately. The CMDs also sensitize the communities where they stay on malaria prevention. They have really helped us health workers. The work load has reduced. We appreciate [their contribution]". *(KI, health worker)

*"There is a good relationship with us because they know where they stop. They know that they can't manage [everything] according to how they were trained. [For complicated cases] they immediately refer them to the health facilities. These people have really helped very much in the areas where they operate. We have not heard cases that a child has died at the CMDs level." *(KI, health worker)

However, there were also some reservations about CMDs performance in the management of malaria at community level. Some informants reported that CMDs are sometimes difficult to find and in some instances they are looked down upon by caregivers because they are not trained professionals. Others reported that medicine distribution is a difficult task and that CMDs can easily give medicines without understanding what the underlying problem is. Some KIs indicated that CMDs are not trusted because of their low level of education.

*"In some cases [instances] they are minimized because they are not trained professionals. Medicine distribution [prescription] is very hard and they can easily give you medicine without understanding what you are suffering from. If you explain to him that you have malaria or that you are not feeling well, of cause; for him he has the medicine he just gives you". *(FGD, male caregivers)

### Opinions, concerns and acceptability of the use of RDTs by CMDs

Although it emerged that community members had some doubts about CMD skills, there was consistency across the FGDs and KIIs a high degree of confidence that CMDs can perform the test once they are provided with adequate training. This trust and confidence was based on the education level of the CMDs, their experience, as well as perceptions about their commitment to work.

*"If a person is trustworthy in a small thing they can work equally well even in a bigger one. These people have worked well and we never got problems with them. If my child falls sick, I just cross the road to him instead of going to Namungalwe where a person from Marina [name of place outside Iganga] knows nothing about me." *(FGD, male caregivers)

Community members also echoed the need for refresher training for CMDs in addition to training on RDTs.

*"That will be very good [introducing RDTs] only that they will need to be trained like how they were trained before starting to give out coartem."(*KI, health worker)

*"I would second it [introducing RDTs] provided they are trained to do it safely. It is very important." *(KI, community leader)

CMDs expressed willingness to use RDTs as part of the services they provide, and are confident they can perform it if they receive adequate training. CMDs contended that they have been voluntarily distributing drugs and would now volunteer also for this new task. CMDs were optimistic that the community would welcome their use of RDTs.

*"If they know that we have been trained, they will accept [the use of RDTs]. Even for the giving out drugs they first resisted but later accepted." *(FGD, CMDs)

Also the majority of health workers supported the idea of CMDs conducting RDTs as long as they are given practical training to acquire additional skills, accurate information, and guidelines to ensure safety. They asserted that this is one way of taking services nearer to people, encourage early treatment seeking behaviour, and is one way of providing CMDs with additional skills crucial for managing malaria. They also appreciated the fact that the CMD treatment of malaria could be performed more effectively if an RDT was introduced, so as to distinguish between malaria and other types of fever.

*"That is a good thing [the use of RDTs], and it will work because people have been coming here a lot because of malaria. So if it [RDT and malaria treatment] is taken near them it will help them so that a person knows what is required and will help them get early treatment. But these CMDs need to be given additional skills because they need to have enough information to avoid questions like why they are taking blood." *(KI, health worker)

### Fears and stigma associated with taking blood from children in the community

Even though most community members were positive towards the use of RDTs by CMDs, many had mixed feelings about the taking of blood from children. The fear included concerns that their children could get infected with HIV in the process of undertaking an RDT and that the blood could be used to test children for HIV rather than malaria.

*"People have fears about blood and that it can lead to contracting HIV. It would also imply to some people that when they prick to test the blood, they think you are testing for HIV but not malaria. They believe you can prove malaria by mere looking at signs like vomiting, shivering, diarrhea and other basic signs." *(KI, community leader)

Another frequently mentioned concern was that the blood could end up in the wrong hands and be used for witchcraft.

*"Since people use it [blood] for bewitching, someone can draw it from you without you being aware that it will be used for other things. If you have a co-wife she can use it to bewitch you to death or stop producing children. Some say that they use it as sacrifice." *(FGD, female caregivers)

### Challenges anticipated to hamper the success of CMD management of fever using RDTs

Given the tasks that the CMDs are given, views regarding the challenges that CMDs are likely to face were solicited. The reported challenges that were foreseen included that CMDs will lack transport for follow up of patients and for replenishment of supplies from the health facilities, that adults will try to force CMDs to also test them for malaria, that caregivers will insist that CMDs treat their children even when the child needs referral, and that some leaders will try to seek for favours for themselves and their relatives.

*"I have seen some of them [CMDs] walking all the way to the health center to get drugs, and when following up children. They need to be assisted with transport for drugs". *(FGD, male caregivers)

*"These tests [RDTs] will create a lot of interest. I am concerned that adults will also demand to be tested. I think also some of our people will insist that their children are treated when they should be referred as now CMDs will be seen as "little doctors" who can take blood and examine it". *(KI, community leader)

## Discussion

The study found that community medicine distributors (CMDs) were trusted by their communities because of their voluntary services, ease of access, and perceived effectiveness of the anti-malarial drugs they use. This is in line with the findings from a multi-country study (including Uganda) which showed high community acceptability of CMD management of sick children, with most children receiving treatment on the same day or within one day of onset of symptoms [39]. Kilian *et al *[13] found similar results in western Uganda, where women showed high acceptability of pre-packaged anti-malarial drugs in the hands of CMDs. However, while Ajayi *et al *[39] also concluded that most caregivers perceived the anti-malarials used by CMDs to be effective, Nsabagasani *et al *[40] demonstrated that although caregivers concurred that they were benefiting from the CMD programme, the treatment was perceived as a drug of lower quality. The contradicting finding is likely to be a result of the type of medicine used, as Ajayi *et al *[39] evaluated the use of highly efficacious pre-packed ACT, whereas Nsabagasani *et al *[40] evaluated the use of Homapak^® ^(chloroquine and sulphadoxine/pyrimethamine) - a combination drug known to have low clinical efficacy due to parasite resistance.

CMDs were trusted by health workers who recognized that the CMDs have contributed to a reduced workload in the health facilities. This concurs with what was found in a rural district in Burkina Faso, where a reduction in workload at peripheral health facilities was observed after HMM was implemented [41]. Similarly, HMM with ACT using community health workers who are supervised regularly was found to be feasible and acceptable by health workers in Ghana [42]. In resource constrained rural communities with geographically sparse health services, CMDs could fill a major gap in access to health care for under-five children [43]. The high level of community acceptability for HMM is likely a result of its convenience, given the fact that malaria risk is often highest in remote rural areas where the quality and coverage of health services is lowest [44].

In this study, the formal education level of CMDs appeared to be a key factor in their acceptability by the community, with CMDs with low education being viewed less positively. This finding needs to be explored further, as it could have major implications on what criteria should be used to select CMDs under home management of malaria (HMM) programmes. Current selection criteria for the community to consider when they choose their CMDs include: age, literacy levels, and gender but different districts and sub-districts may however, choose different criteria for nominating CMDs and ability to read and write may not be prioritized in certain areas [45]. If shown to be consistent across countries and programmes, this could have negative implications if RDTs are to be deployed. Alternatively, programs that want to scale up CMD use of RDTs would need to demonstrate that CMDs are able to use the test and that communities accept their services, regardless of education level.

A majority of community members, health workers and CMDs welcomed the use of RDTs by CMDs, provided that they are properly trained in their use. A similar study in Sudan also found community acceptability of Coartem^® ^and RDTs in HMM to be high, with a marked increase in treatment-seeking behaviour following the implementation [46]. In the present study, the high community acceptability of RDTs was partly due to the appreciation that CMDs would be able to offer treatment which was based on test results rather than just presence of symptoms and signs.

While RDTs have the potential to greatly enhance the management of febrile illness [47] the success of their use in the hands of CMDs is highly dependent on how the community will accept the services provided. Health care policies such as HMM are often formulated at national level and need to take into account local cultural context; sometimes assumptions are made based on what can be communicated through simplified biomedical terms, and how this will result in behaviour change [48]. However, acceptability of medical care by different ethnic groups is associated with the degree to which the services meet cultural values, norms, and expectations [49]. These factors need to be taken into account to ensure successful uptake of community programs [50]. While a desirable, participatory process in every village is not compatible with rapid national scale-up - a balance needs to be reached [40]. Having CMDs of the same ethnic group, who speak the same language, and are familiar with the culture and its traditions and values are factors that can increase acceptability [51]. The findings of this study also show that the community demands for well trained CMDs to handle and interpret the tests - some critical characteristics also identified by Din and Bell [52]. Equally, programmes will need to address concerns about children with false negative results, as now CMDs will be required to without the ACT. If, however, the child is not followed closely and gets worse or even dies, this could potentially undermine the credibility of the entire programme. In the trial that followed this study, blood slides for microscopy were collected from each child on the same day; microscopy results for all RDT negative children were returned to the CMD within 24 hours to try and address this problem. This approach will not be possible in non-research settings.

Fears and stigma of taking blood of children in the community were common, relating both to risk of exposure to HIV and witchcraft. Myths about blood could have serious consequences to the HMM programme, especially if there is a bad outcome for a child. In Tanzania, though there was a high degree of provider and caregiver acceptance and satisfaction with RDTs, some caregivers had reservations about RDTs, with some thinking they were HIV test kits [30]. To counter for this potential threat to the programme, a well-designed behaviour change communication strategy will be required, since a successful HMM strategy relies not only on adequately trained CMDs and the availability of drugs, but also on a well informed community [53,43].

In this study, community members anticipated that CMDs would face difficulties with adults demanding to be tested and caregivers insisting that their children should be treated instead of being referred. Compliance to guidelines is often a major challenge and a study in Burkina Faso found very low health worker compliance to negative RDT results, as they still went on to prescribe anti-malarial drugs for patients who tested negative [54]. This was also observed in another study in Sudan [46]. These issues will require special attention through frequent supportive supervision meetings to monitor and assess performance, and during regular CMD mentoring sessions. It has been observed that lower level health workers are more likely to comply with guidelines as compared to more experienced or high cadre health workers [55]. A follow up study is planned to assess community acceptability of RDT use by CMDs following RDT deployment in HMM.

## Methodological considerations

One of the limitations of qualitative methods is that the magnitude of a variable of interest across different categories of respondents cannot be measured. However, it was not the intent of this study to quantify the issues under study as a household survey is planned for this purpose. Triangulation of information from caregivers, health providers, CMDs and community and opinion leaders using both FGDs and KIIs, was a useful strategy for checking consistency and contradictions across and within groups [56,57]. The geographical spread of the FGDs allowed us to interview participants from across the study area, getting as close to representative views of the study population as practically possible. Separating women from men in the FGDs and focused questions to the research issues could have promoted free expression of the participants and also reduced answers given to please the researcher.

## Conclusion

Use of rapid diagnostic tests (RDTs) by community medicine distributors (CMDs) is likely to be acceptable by community members given that CMDs are properly trained, supported and provided logistical support. A well-designed behaviour change communication strategy is needed to address the anticipated programmatic challenges as well as community fears and stigma about drawing blood. Level of formal education may have to be a criterion for CHW selection into programmes deploying RDTs.

## Competing interests

The authors declare that they have no competing interests.

## Authors' contributions

DM, JT, JK, GP, PW, FB, BM, HC, GO and KK took part in designing the study, in tools development, in data analysis and in manuscript writing. DM, JK, FB, BM, GO and KK did field work. All authors approved the final manuscript.

## References

[B1] World Health Organization World Malaria Report 2008 2008 WHO Press Geneva

[B2] Black RE Morris SS Bryce J Where and why are 10 million children dying every year? Lancet 2003 361 2226 2234 10.1016/S0140-6736(03)13779-8 12842379

[B3] World Health Organization World Health Statistics Country Health System Fact Sheet Uganda 2006 http://www.afro.who.int/home/countries/fact_sheets/uganda.pdf (Accessed September 19, 2009)

[B4] Rutebemberwa E Kallander K Tomson G Peterson S Pariyo G Determinants of delay in care-seeking for febrile children in eastern Uganda Trop Med Int Health 2009 4 1 8 10.1111/j.1365-3156.2009.02237.x 19222823

[B5] Haines A Sanders D Lehmann U Rowe AK Lawn JE Jan S Walker DG Bhutta Z Achieving child survival goals: potential contribution of community health workers Lancet 2007 369 2121 2131 10.1016/S0140-6736(07)60325-0 17586307

[B6] Pagnoni F Convelbo N Tiendrebeogo J Cousens S Esposito F A community-based program to provide prompt and adequate treatment of presumptive malaria in children Trans R Soc Trop Med Hyg 1997 91 512 517 10.1016/S0035-9203(97)90006-7 9463653

[B7] Kidane G Morrow RH Teaching mothers to provide home treatment of malaria in Tigray, Ethiopia: a randomised trial Lancet 2000 356 550 555 10.1016/S0140-6736(00)02580-0 10950232

[B8] Sirima SB Konate A Tiono AB Convelbo N Cousens S Pagnoni F Early treatment of childhood fevers with pre-packaged antimalarial drugs in the home reduces severe malaria morbidity in Burkina Faso Trop Med Int Health 2003 8 133 139 10.1046/j.1365-3156.2003.00997.x 12581438

[B9] Ministry of Health Uganda Implementation guidelines for the Home Based Management of fever strategy 2002 1 MoH/UNICEF/BASICS/DISH, Kampala, Uganda

[B10] Young MW Effective Management of Childhood Malaria at the Community Level: Program Experience to Guide the Research Agenda Paper for the WHO/TDR Scientific Working Group on Malaria, Geneva, Switzerland, 2003, 24-27 March

[B11] Kallander K Nsungwa-Sabiiti J Home-based management of malaria in the era of urbanization Lancet 2009 373 1582 1584 10.1016/S0140-6736(09)60359-7 19362362

[B12] Ministry of Health, Uganda Home based management of fever strategy in Uganda: survey report 2004 WHO, BASICS

[B13] Kilian AHD Tindyebwa D Gulck T Byamukama T Rubaale T Kabagambe G Korte R Attitude of women in western Uganda towards pre-packed, unit-dosed malaria treatment for children Trop Med Int Health 2003 8 431 438 10.1046/j.1365-3156.2003.01044.x 12753639

[B14] D'alessandro U Talisuna A Boelaert M Editorial: Should Artemisininbased combination treatment be used in the home-based management of malaria? Trop Med Int Health 2005 10 1 2 10.1111/j.1365-3156.2004.01375.x 15655007

[B15] Staedke GS Mwebaza N Kamya RM Clark TD Dorsey G Rosenthal PJ Whitty CJM Home management of malaria with artemether-lumefantrine compared with standard care in urban Ugandan children: a randomized controlled trial Lancet 2009 373 1623 1631 10.1016/S0140-6736(09)60328-7 19362361

[B16] Barnes KI Durrheim ND Little F Jackson A Mehta U Allen E Dlamini SS Tsoka J Bredenkamp B Mthembu JD White NJ Sharp BL Effect of artemether-lumefantrine policy and improved vector control on malaria burden in KwaZulu-Natal, South Africa PLoS Med 2005 2 e330 10.1371/journal.pmed.0020330 16187798 PMC1240068

[B17] Nyarango PM Gebremeskel T Mebrahtu G Mufunda J Abdulmumini U Ogbamariam A Kosia A Gebremichael A Gunawardena D Ghebrat Y Okbaldet Y A steep decline of malaria morbidity and mortality trends in Eritrea between 2000 and 2004: the effect of combination of control methods Malar J 2006 5 33 10.1186/1475-2875-5-33 16635265 PMC1501031

[B18] Bhattarai A Ali AS Kachur PS Mårtensson A Abbas AK Khatib R Al-mafazy A Ramsan M Rotllant G Gerstenmaier JF Molteni F Abdulla S Montgomery SM Kaneko Björkman A Impact of artemisinin-based combination therapy and insecticide-treated nets on malaria burden in Zanzibar PLoS Med 2007 4 e309 10.1371/journal.pmed.0040309 17988171 PMC2062481

[B19] Otten M Aregawi M Were W Karema C Medin A Bekele W Jima D Gausi K Komatsu R Korenromp E Low-Beer D Grabowsky M Initial evidence of reduction of malaria cases and deaths in Rwanda and Ethiopia due to rapid scale-up of malaria prevention and treatment Malar J 2009 8 14 10.1186/1475-2875-8-14 19144183 PMC2653503

[B20] World Health Organization World Malaria Report 2009 Geneva: WHO Press

[B21] D'Acremont V Lengeler C Mshinda H Mshinda H Mtasiwa D Tanner M Blaise Genton B Time To Move from Presumptive Malaria Treatment to Laboratory-Confirmed Diagnosis and Treatment in African Children with Fever PLoS Med 2009 6 e252 10.1371/journal.pmed.0050252 19127974 PMC2613421

[B22] Reyburn H Mbatia R Drakeley C Carneiro I Mwakasungula E Mwerinde O Saganda K Shao J Kitua A Olomi R Greenwood BM Whitty CJM Overdiagnosis of malaria in patients with severe febrile illness in Tanzania: A prospective study BMJ 2004 329 1212 10.1136/bmj.38251.658229.55 15542534 PMC529364

[B23] President's Malaria Initiative Uganda Malaria Operational Plan for FY2010 2009

[B24] Moody A Rapid Diagnostic Tests for Malaria Parasites Clin Microbiol Rev 2002 15 66 78 10.1128/CMR.15.1.66-78.2002 11781267 PMC118060

[B25] Murray CK Bell D Gasser RA Wongsrichanalai C Rapid diagnostic testing for malaria Trop Med Int Health 2003 8 876 883 10.1046/j.1365-3156.2003.01115.x 14516298

[B26] Bell D Wongsrichanalai C Barnwell JW Ensuring quality and access for malaria diagnosis: how can it be achieved? Nat Rev Microbiol 2006 4 682 695 10.1038/nrmicro1474 16912713

[B27] Harvey A Jennings L Chinyama M Masaninga F Mulholland K Bell D Improving community health worker use of malaria rapid diagnostic tests in Zambia: package instructions, job aid and job aid-plus-training Malar J 2008 7 160 10.1186/1475-2875-7-160 18718028 PMC2547110

[B28] Hildenwall H Lindkvist J Tumwine JK Bergqvist Y Pariyo G Tomson G Peterson S Low validity of caretakers' reports on use of selected antimalarials and antibiotics in children with severe pneumonia at an urban hospital in Uganda Trans R Soc Trop Med Hyg 2009 103 95 101 10.1016/j.trstmh.2008.04.046 18678381

[B29] Drakeley C Reyburn H Out with the old, in with the new: the utility of rapid diagnostic tests for malaria diagnosis in Africa Trans R Soc Trop Med Hyg 2009 103 333 337 10.1016/j.trstmh.2008.10.003 19019399

[B30] Williams HA Causer L Metta E Malila A O'Reilly T Salim Abdulla S Kachur PS Bloland PB Dispensary level pilot implementation of rapid diagnostic tests: an evaluation of RDT acceptance and usage by providers and patients - Tanzania Malar J 2008 7 239 10.1186/1475-2875-7-239 19019233 PMC2613413

[B31] Fairhead J Leach M Small M Where techno-science meets poverty: Medical research and the economy of blood in the Gambia, West Africa Soc Sci Med 2006 63 1109 1120 10.1016/j.socscimed.2006.02.018 16630676

[B32] Geissler PW Pool R Editorial: Popular concerns about medical research projects in sub-Saharan Africa - a critical voice in debates about medical research ethics Trop Med Int Health 2006 11 975 982 10.1111/j.1365-3156.2006.01682.x 16827698

[B33] Newton S Doku V Geissler W Asante KP Cousens S Drawing blood from young children: lessons learned from a trial in Ghana Trans R Soc Trop Med Hyg 2009 103 497 499 10.1016/j.trstmh.2008.11.030 19155032

[B34] English M Reyburn H Goodman C Snow RW Abandoning presumptive antimalarial treatment for febrile children aged less than five years--A case of running before we can walk? PLoS Med 2009 6 e1000015 10.1371/journal.pmed.1000015 19127977 PMC2613424

[B35] Berwick DM Disseminating innovations in health care JAMA 2003 289 1969 1975 10.1001/jama.289.15.1969 12697800

[B36] Netmark Regional Africa program briefing book; Insecticide treated materials in Uganda 2000 http://www.netmarkafrica.org/countries/uganda/briefbook/uganda_briefing_book.pdf (Accessed September 19, 2009)

[B37] Rutebemberwa E Nsabagasani X Pariyo G Tomson G Peterson S Kallander K Use of drugs, perceived drug efficacy and preferred providers for febrile children: implications for home management of fever Malar J 2009 8 131 10.1186/1475-2875-8-131 19523220 PMC2702349

[B38] Graneheim H Lundman B Qualitative content analysis in nursing research: concepts, procedures and measures to achieve trustworthiness Nurse Educ Today 2004 24 105 112 10.1016/j.nedt.2003.10.001 14769454

[B39] Ajayi IO Browne EN Garshong B Bateganya F Yusuf B Agyei-Baffour P Doamekpor L Balyeku A Munguti K Counsens S Nanyunja M Pagnoni F Feasibility and acceptability of artemisinin-based combination therapy for the home management of malaria in four African sites Malar J 2008 7 6 10.1186/1475-2875-7-6 18182114 PMC2254635

[B40] Nsabagasani X Nsungwa-Sabiiti J Kallander K Peterson S Pariyo G Tomson G Home based management of malaria in rural Uganda: Community perceptions and provider opinions Malar J 2007 6 11 10.1186/1475-2875-6-11 17257396 PMC1797180

[B41] Tiono A Kaboré Y Traoré A Convelbo N Pagnoni F Sirima S Implementation of Home based management of malaria in children reduces the work load for peripheral health facilities in a rural district of Burkina Faso Malar J 2008 7 201 10.1186/1475-2875-7-201 18834504 PMC2570683

[B42] Chinbuah M Gyapong O Pagnoni F Wellington K Gyapong M Feasibility and acceptability of the use of artemether-lumefantrine in the home management of uncomplicated malaria in children 6-59 months old in Ghana Trop Med Int Health 2006 11 1003 1116 10.1111/j.1365-3156.2006.01654.x 16827701

[B43] Pagnoni F Home management of malaria Lancet 2009 374 288 289 10.1016/S0140-6736(09)61358-1 19632486

[B44] Bhasin VK Nair L ACT now - with caution - for malaria treatments Lancet Infect Dis 2003 3 609 10.1016/S1473-3099(03)00767-9 14522258

[B45] Ministry of Health, Uganda Integrated community case management of childhood malaria, pneumonia and diarrhea 2010 Implementation Guide

[B46] Elmardi A Malik M Abdelgadir T Ali SH Elsyed AH Mudather MA Elhassan AH Adam I Feasibility and acceptability of home-based management of malaria strategy adapted to Sudan's conditions using artemisinin-based combination therapy and rapid diagnostic test Malar J 2009 8 39 10.1186/1475-2875-8-39 19272157 PMC2660358

[B47] Bell D Moody A Malaria Rapid Diagnostic Tests: One Size May Not Fit All Clin Microbiol Rev 2002 15 771 772 10.1128/CMR.15.4.771-772.2002 12364379 PMC126861

[B48] Geest VDS Is there a role for traditional medicine in basic health services in Africa? A plea for a community perspective Trop Med Int Health 1997 2 903 911 10.1046/j.1365-3156.1997.d01-410.x 9315049

[B49] Etkin NL van der Geest S, Whyte SR Cultural constructions of efficacy The context of medicines in developing countries 1991 Amsterdam: Het Spinhuis Publishers

[B50] Patterson AE Winch P Gilroy K Doumbia S Local terminology for medicines to treat fever in Bougouni District, Mali: implications for the introduction and evaluation of malaria treatment policies Trop Med Int Health 2006 11 1613 1624 10.1111/j.1365-3156.2006.01713.x 17002736

[B51] Cox C Ephross P Ethnicity and social work practice 1996 Oxford University Press

[B52] Dini L Bell D Malaria rapid diagnostic tests: A revolution and challenge for management of febrile disease CME 2008 26 297 298

[B53] Charlwood D The paradox of home management of malaria with artemisinin combinations Trends Parasitol 2004 20 405 406 10.1016/j.pt.2004.07.006 15324729

[B54] Bisoffi Z Gobbi F Angheben A Van den Ende J The role of rapid diagnostic tests in managing malaria PLoS Med 2009 6 e1000063 10.1371/journal.pmed.1000063 19399160 PMC2667642

[B55] Zurovac D Rowe AK Ochola SA Noor AM Midia B English M Snow RW Predictors of the quality of health worker practices for uncomplicated malaria at government health facilities in Kenya Int J Epidemiol 2004 33 1080 1091 10.1093/ije/dyh253 15256523

[B56] Flick U Triangulation revisited: strategy of validation or alternative? Journal for the Theory of Social Behaviour 1992 22 175 197 10.1111/j.1468-5914.1992.tb00215.x

[B57] Berg BL Qualitative research methods for social sciences 2001 Needham Heights: Allyn and Bacon

